# Human bone marrow mesenchymal progenitors: perspectives on an optimized *in vitro* manipulation

**DOI:** 10.3389/fcell.2014.00007

**Published:** 2014-03-27

**Authors:** Eric Cordeiro-Spinetti, Wallace de Mello, Lucas Siqueira Trindade, Dennis D. Taub, Russell S. Taichman, Alex Balduino

**Affiliations:** ^1^LaBioTeC, Universidade Veiga de AlmeidaRio de Janeiro, Brazil; ^2^Laboratório de Pesquisas sobre o Timo, Instituto Oswaldo CruzFiocruz, Rio de Janeiro, Brazil; ^3^Department of Biological Sciences, Tokyo Metropolitan UniversityHachioji, Tokyo, Japan; ^4^Department of Vetarans Affairs, Hematology and Immunology Research, Washington DC Veterans Affairs Medical CenterWashington, DC, USA; ^5^School of Dentistry, Department of Periodontics and Oral Medicine, University of MichiganAnn Arbor, MI, USA; ^6^Excellion Serviços BiomédicosPetrópolis, Rio de Janeiro, Brazil

**Keywords:** bone marrow, mesenchymal stem cell, colony, *in vitro* expansion, mutipotent progenitor

## Abstract

When it comes to regenerative medicine, mesenchymal stem cells (MSCs) are considered one of the most promising cell types for use in many cell therapies and bioengineering protocols. The International Society of Cellular Therapy recommended minimal criteria for defining multipotential MSC is based on adhesion and multipotency *in vitro*, and the presence or absence of select surface markers. Though these criteria help minimize discrepancies and allow some comparisons of data generated in different laboratories, the conditions in which cells are isolated and expanded are often not considered. Herein, we propose and recommend a few procedures to be followed to facilitate the establishment of quality control standards when working with mesenchymal progenitors isolation and expansion. Following these procedures, the classic Colony-Forming Unit-Fibroblast (CFU-f) assay is revisited and three major topics are considered to define conditions and to assist on protocol optimization and data interpretation. We envision that the creation of a guideline will help in the identification and isolation of long-term stem cells and short-term progenitors to better explore their regenerative potential for multiple therapeutic purposes.

## Introduction

To minimize discrepancies and inconsistencies, and allow comparison of data generated in different laboratories, members of the International Society of Cellular Therapy (ISCT) (Horwitz et al., 2005) have recommended minimal criteria for defining multipotential mesenchymal stem cells (MSCs). By ISCT criteria, MSCs must adhere and grow on a substrate *in vitro* and give rise to osteoblasts, chondrocytes, adipocytes, and hematopoiesis-supporting reticular stroma when cultured under proper differentiation conditions. MSCs must also express CD73, CD90, and CD105, but not express hematopoietic cells and endothelial cells markers (Barry et al., 1999, 2001; Jones et al., 2002; Horwitz et al., 2005; Dominici et al., 2006; Sarugaser et al., 2009). Further investigation unveiled a few other surface markers, among which CD146 has been demonstrated to be consistently expressed by all MSCs and progenitors (Bianco et al., 1988; Shih, 1999; Dennis et al., 2002; Tuli et al., 2003; Zannettino et al., 2003; Sacchetti et al., 2007).

Growing evidence indicates an intimate relationship between MSCs and those cells identified as pericytes, since these two populations demonstrate similar behavior and potential *in vitro* and *in vivo* (Shi and Gronthos, 2003; Sacchetti et al., 2007; Taichman et al., 2010; Péault, 2012). Pericytes are perivascular cells which reside on the abluminal side of sinusoids and are known to express the proteoglycan NG2, alpha smooth muscle actin (αSMA), and Platelet Derived Growth Factor Receptor (PDGFR) (Andreeva et al., 1998; Crisan et al., 2008a, 2009; Maier et al., 2010). Similarities to pericytes led to the concept that all tissues in the body harbor their own population of mesenchymal*-like* stem cells. Of note, it is important to stress that these cells are influenced by the niche they occupy *in vivo*, making them similar to each other, but with a few distinct characteristics and differentiation bias. Mesenchymal-*like* stem cells and progenitors have been isolated from several tissues, but adipose tissue and bone marrow are usually indicated as most promising sources of these cells by those working in the cell therapy and bioengineering fields (Da Silva Meirelles et al., 2006; Crisan et al., 2008b; Corselli et al., 2011). Yet to date, no specific or combination of markers can be used to distinguish multipotential MSCs from committed progenitors. A differentiation cascade, similar to the hematopoietic system, has not yet been assembled and confirmed.

Friedenstein and coworkers (Friedenstein et al., 1974a,b; Friedenstein, 1976; Owen and Friedenstein, 1988) were the first to describe the existence of a second category of progenitors residing in the marrow cavity, and named them stromal progenitor cells. His cues came with an *in vivo* assay, in which bone marrow cells were loaded into chambers and implanted subcutaneously in rats (Friedenstein et al., 1966, 1974b). After several weeks of implantation, bone*-like* mineralized nodules and cuboidal osteoblasts were observed inside the chambers in the new-formed tissue. The chamber's pores were too small and prevented cells from migrating into or out of the chambers, supporting the concept that the new bony tissue formed inside the chambers was exclusively generated from donor cells rather than recipient cells. Additional studies identified this activity belonging to the non-hematopoietic stromal fraction. *In vitro*, they showed that when bone marrow cells were placed into culture at low density, a few of them adhered, proliferated, and gave rise to colonies of fibroblast*-like* cells (CFU-f). These adherent fibroblast*-like* cells, but not the hematopoietic cells, when implanted *in vivo*, differentiated into bone tissue and bone marrow stroma, confirming that the bone marrow microenvironment is the niche for two distinct progenitors populations (Friedenstein et al., 1966, 1974a; Owen and Friedenstein, 1988). Cells were then named stromal stem cells. Later, further clonal manipulation and *in vivo* observations led different authors to propose different names, such as mesenchymal stem cells (Caplan, 1991, 2007) and skeletal stem cells (Bianco, 2011), to define almost the same cell population. However, it is important to stress that, even though these names have been used unrestrictedly as synonyms by several different authors, conceptually and originally, they indicate significant differences among the cells, mainly concerning their differentiation potential.

Although several research groups have described different strategies to isolate mesenchymal cells, the CFU-f assay has undergone almost no change since its original description by Friedenstein and coworkers (Friedenstein et al., 1970, 1974b). Higher proliferative rates are usually related to the stem cell and progenitor populations in most normal tissues. It is therefore assumed that each colony of fibroblast*-like* cells (CFU-f) originates from a single stem and/or progenitor cell (Friedenstein et al., 1966, 1974a,b; Latsinik and Epikhina, 1974; Friedenstein, 1976), and the number of colonies observed represents the number of mesenchymal progenitors as a fraction of the number of nucleated cells plated.

For researchers working with mesenchymal cells isolation and expansion, this is the most widely accepted assay used to quantify progenitors numbers. In the present perspective we addressed major topics we believe are most relevant regarding a few specific and distinct aspects of *in vitro* cell adhesion and growth. Even though CFU-f assay is very simple to perform, we proposed three different strategies based on progenitors *in vitro* clonogenic potential, which might be helpful to define standard conditions to optimize *in vitro* manipulation, and provide data linearity and reproducibility.

## Number of cells in a colony

**Figure 1:**
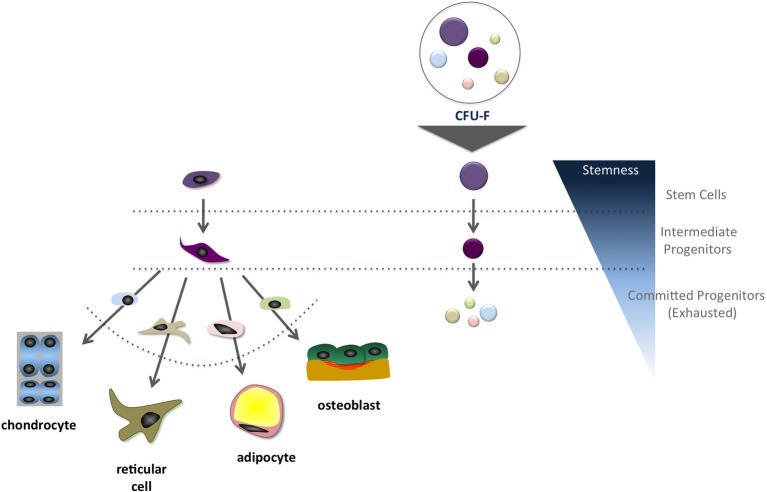
**Schematic representation of the suggested correlation between the mesenchymal stem cell differentiation cascade and colony size *in vitro*.** It is frequently inferred that most primitive progenitors give rise to larger colonies compared to those originated from intermediate and committed progenitors.

In the late 1960's and early 1970's, Alexander Friedenstein and colleagues (Friedenstein et al., 1966, 1970) began their journey into the bone marrow cavity and defined the primary conditions to quantify a sub-population of, by that time, osteogenic progenitors among all bone marrow stromal cells (Friedenstein et al., 1966, 1982). In the original protocol, single cell suspensions of bone marrow cells are plated at low-density (10^4^–10^5^ nucleated cells per cm^2^) and incubated in DMEM supplemented with fetal bovine serum (FBS). Seventy-two hours later, the non-adherent cells are washed out and the adherent fraction is incubated in fresh culture medium. Culture medium is renewed every 3–4 days over a ten-day culture. After a total of 13 days, the cells are fixed and further stained in crystal violet, and the colonies are counted (Satomura et al., 2000; Kuznetsov et al., 2009). It is assumed in this case that when bone marrow cells are plated in low-density cultures, the colonies will not reach each other's borders supporting that each colony is derived from a single progenitor. However, it is important to keep in mind that colonies are heterogeneous and, although each one is derived from a single progenitor, not all display a multilineage differentiation potential. Many are already committed to a specific lineage, following the hierarchical*-like* and controlled differentiation cascade (Muraglia et al., 2000; Sarugaser et al., 2009; Russell et al., 2010). Colonies also display different sizes and cell distribution within the cultures, which may correlate to cell differentiation stage (Figure 1). Additional studies have revealed that ~30% of all BM mesenchymal progenitors colonies present trilineage potential—osteogenic, chondrogenic, and adipogenic—*in vitro*. Sacchetti and coworkers (Sacchetti et al., 2007) demonstrated that ~50% of the CD146^+^ clonal mesenchymal progenitors isolated from the bone marrow cultures give rise to compact bone, but not bone marrow, when implanted *in vivo*, indicating that half of the progenitors, upon isolation from an adult bone marrow, are already committed to the osteogenic lineage.

As for several tissues, cells are classified into three categories: (1) stem cells, (2) intermediate progenitors and (3) differentiated cells. Typically, differentiated cells possess low proliferation ability *in vitro*, while intermediate progenitors present high proliferative rates under stimulus. On the other hand, stem cells are quiescent cells *in vivo*, but *in vitro*, under the proper culture conditions, exit the quiescence stage and become highly proliferative (Stanley et al., 1971; Urabe et al., 1979; Nicola and Metcalf, 1986; Oh and Humphries, 2012; Bianco et al., 2013). When analyzed in this perspective, it is expected that several stromal cell populations adhere to the culture flask surface in the first three days of culture, namely differentiated reticular cells, committed progenitors and stem cells. Only progenitors and stem cells will proliferate to generate colonies. Regardless progenitors commitment, what features each group of cells must present to be identified as a colony? Based on the original protocol and refinements suggested by several laboratories (Wagner et al., 2009; Bianco et al., 2013), only colonies consisting of more than 50 cells should be classified as a colony (Kuznetsov et al., 2009). Colonies quantification can be performed under the microscope, but stained colonies with more than 50 cells are easily observed directly by the “naked eye.” It must be acknowledged that growth-promoting activity will vary from FBS lot to lot, which may change CFU-f results. Thus, a quality control should be used to screen FBS lots to avoid suboptimal or “superoptimal” conditions, which may result in changes in colony formation and impact cell growth and differentiation (Mannello and Tonti, 2007).

**Figure 2:**
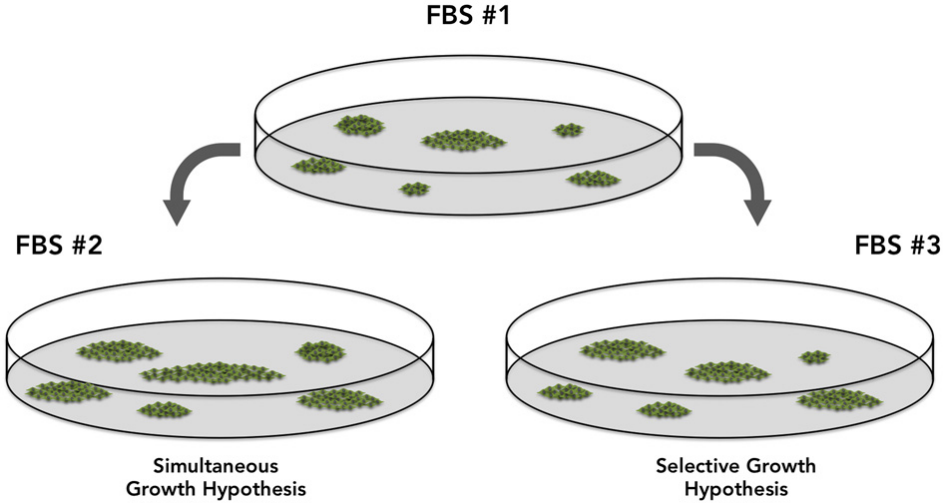
**Two hypotheses discussing the potential differences in the Colony Forming Efficiency results associated with the usage of different fetal bovine serum lots.** The “Simultaneous Growth Hypothesis” suggests colonies are similarly affected and respond simultaneously, proliferating more or less, but in the same proportion. The “Selective Growth Hypothesis,” suggests that in response to different FBS lots, a few progenitors will proliferate more than others, as a result of growth factors concentration and combination.

It is clear that cell proliferation status changes accordingly as FBS is changed, which will considerably impact CFU-f results. Cell proliferation depends upon growth factors concentration and, regardless all the controls applied to serum fabrication, this varies largely from FBS lot to lot. Conversely, it is not clear how progenitors respond to serum variations. It must be determined if proliferation of progenitors *in vitro* follows a simultaneous or selective growth pattern, the latter being all progenitors will respond differently when FBS lot is changed (Figure 2). It is expected that all progenitors in a plate respond similarly (Simultaneous Growth Hypothesis) and, as the FBS lot is changed, they all may proliferate more or less. It is unclear if this is what occurs *in vitro*. Different from the concept that all colonies will be bigger or smaller, the Selective Growth Hypothesis proposes that big colonies might get bigger and small clusters might even not reach the colony status.

At this point, one should know that colony size (the number of cells in a colony) will matter, but only under optimized conditions. To avoid discrepancies and ensure reproducibility, the ideal condition would be to run the CFE assay under chemically defined and controlled culture medium conditions, using recombinant growth factors instead of serum, but this can be cost prohibitive. Moreover, what combination of factors is necessary to expand mesenchymal progenitors *in vitro* remains to be defined. We propose, however, that colony number, colony size, and progenitors phenotype be tested every time serum change is necessary.

## Does colony size relate to stemness?

Colony forming ability is not exclusive to the mesenchymal system (Queensberry et al., 1974; Dexter, 1979; Nicola and Metcalf, 1986). A similar *in vitro* assay has been widely used to quantify hematopoietic progenitor cells. In the past, this was the only quantification tool, but it was replaced by the development of more meticulous flow cytometric phenotyping methods, which is now mostly utilized to quantify and identify hematopoietic stem cells and progenitors, although the colony assay remains widely used. It must be clear, however, that cell transplantation into myeloablated animals is the only way to fully identify *bona fide* long-term hematopoietic stem cells (Morrison et al., 1995; Gazit et al., 2008).

Hematopoietic stem cells and progenitor colony forming ability is observed when cells are cultured in semi-solid culture medium, which maintains them in close proximity to each other, as they proliferate in the presence of specific growth factors (Queensberry et al., 1974; Dexter, 1979; Nicola and Metcalf, 1986). Unlike the CFU-f assay, which is used to quantify MSCs and committed progenitors indiscriminately, the colony forming assay can be used to quantify lymphoid, erythroid, myeloid, and multipotent progenitors separately depending on the combination of specific growth factors added to the culture system (Stanley et al., 1971; Dexter, 1979; Urabe et al., 1979; Nicola and Metcalf, 1986; Quesenberry et al., 1987). Hematopoietic progenitors grow at different culture rates and present distinct morphologies *in vitro*, allowing identification and quantification of different progenitors separately and quickly.

One major observation from the hematopoietic colony-forming assay is that committed progenitors start to proliferate early (day 1 of culture), and develop into distinct colonies in 5–10 days. On the other hand, most primitive progenitors can take a few days to exit the quiescent stage and will eventually form full colonies after 9–14 days in culture (Stanley et al., 1971; Dexter, 1979; Urabe et al., 1979; Nicola and Metcalf, 1986; Quesenberry et al., 1987). Although primitive progenitors take longer to form colonies, the colonies generated are typically 2–4 times larger than those derived from committed progenitors. This *in vitro* behavior is in full accordance with *in vivo* observations, as hematopoietic stem cells are typically quiescent however, when properly stimulated, achieve higher proliferative rates compared to other progenitors (Carow et al., 1993; Ponchio et al., 1995; Hao et al., 1996; Petzer et al., 1996; Oh and Humphries, 2012). For these reasons it is often inferred that the larger the colony, the more primitive the progenitor it had been derived from.

A similar line of observation could be applied to the colony-forming unit fibroblast assay. In a 13-day culture, the committed progenitors will initiate proliferation faster *in vitro* giving rise to colonies, but these cells will rapidly undergo clonal exhaustion. Conversely, uncommitted progenitors will take longer to activate the proliferation cascade, and do not arrest during the culture period and thus result in larger colonies. Indeed, it is not unusual to correlate colony size with primitiveness of the cell of origin.

**Figure 3:**
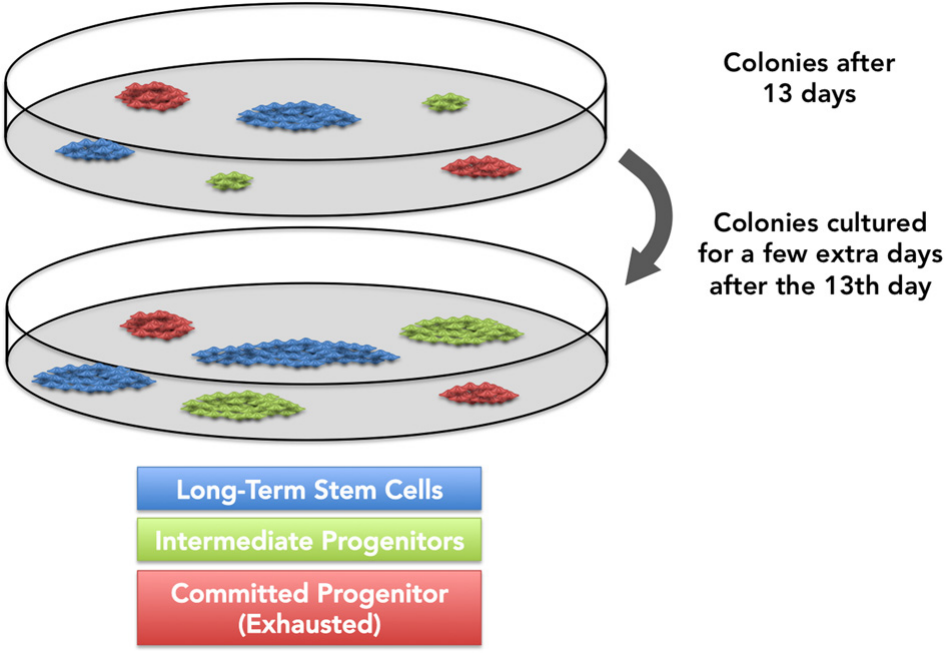
**It is generally accepted that most primitive progenitors, upon exiting the lag growth phase, will reach higher proliferation rates than committed progenitors.** However, recent data indicate that a few progenitors might require a longer time to enter replicative cycles *in vitro*. We and others hypothesize that, if cultured longer, these progenitors might originate larger colonies.

A question remains unanswered: “Is thirteen days sufficient for all multipotential stem cells to enter the cell cycle and become highly proliferative cells *ex vivo*?” Primitive stem cells are likely to remain deeply quiescent or proliferate very slowly *in vivo*. When placed *in vitro*, they may require longer to leave the quiescent stage but, once stimulated, their proliferative rate is likely to increase beyond that observed for intermediate progenitors. Many hypothesize that the most quiescent mesenchymal stem cells might take longer to start proliferation *in vitro*, which means that a few small/medium colonies may not be representative of exhausted committed progenitors, but rather indicate yet-to-proliferate stem cells (Figure 3). This possibility challenges many assumptions derived from the CFU-f assay, and suggests that colony size may not necessarily reflect primitiveness. Cells in colonies derived from committed progenitors are usually large stellate*-like* cells, while in colonies derived from stem cells are often small and fusiform (Gothard et al., 2013). In this case, cell morphology may be a useful tool to tell apart exhausted or expanding small- and medium-sized colonies.

As discussed above, growth factor concentrations in FBS differs among serum lots, and does have significant outcomes on CFU-f size, frequency and even colony morphology. Viewed in terms of the Selective Growth Hypothesis (Figure 2), distinct categories of mesenchymal progenitors may be dependent upon different growth factors (and concentrations) to proliferate (or remain quiescent) *in vitro*.

## Progenitors adhesion *in vitro*

Previously published data imply that all human bone marrow-derived mesenchymal stem cells and progenitors with the ability to form fibroblast*-like* cell colonies adhere within 2–48 h *in vitro*, followed by a fetal bovine serum-dependent growth (Friedenstein, 1976; Castro-Malaspina et al., 1980; Kastrinaki et al., 2008; Kuznetsov et al., 2009). After this period of incubation, non-adherent cells are discarded and adherent cells are further expanded for at least 10 additional days. The original protocol designed by Friedenstein and coworkers, and corroborated by several other laboratories (Friedenstein et al., 1966, 1970; Owen and Friedenstein, 1988), established the requirement for a 72-h adhesion period prior to the elimination of non-adherent cells. This adhesion period is still considered a critical step for isolation and expansion of these progenitors. It has been described that bone marrow stromal cells must adhere to a substrate *in vitro* for their survival and proliferation, regardless their differentiation potential (Bruder et al., 1997; Pittenger et al., 1999; Dominici et al., 2006). Notwithstanding, authors demonstrate that stromal progenitor cells, or at least a subset of them, can be maintained in stirred suspension cultures for 21 days, and might even proliferate when induced by a combination of cytokines and growth factors (Baksh et al., 2003). Which specific stromal progenitors subsets remain in suspension are yet to be determined.

The average colony forming efficiency for normal human adult bone marrow may vary from 1 to 30 per 1 × 10^5^ nucleated marrow cells (Beresford et al., 1994; Oreffo et al., 1998; Doucet et al., 2005; Bernardo et al., 2007; Kuznetsov et al., 2009). As previously discussed (Kuznetsov et al., 2009), such different values might be the result of either distinct cell isolation/preparation procedures or cell culture conditions, the latter meaning FBS capacity to induce progenitors proliferation *in vitro*. Although proliferation status has been a very useful tool to evaluate FBS quality, progenitors adhesion capacity, not usually considered, is crucial for colony forming efficiency and does affect the results. It is not clear, however, if and how different concentrations of growth factors and cytokines in FBS would impact progenitors adhesion *in vitro*. Most recently, Di Maggio and coworkers (Di Maggio et al., 2012) demonstrated that highly proliferative multipotent progenitors can be isolated from the non-adherent fraction, after 3 days, when bone marrow cells are cultured in the presence of FGF2. In addition to previous observations (Baksh et al., 2003), these data indicate that mesenchymal progenitors, or at least a subset, might change its adhesion properties accordingly as growth factors and cytokines concentration change in FBS. Currently, it is difficult to precise how much time mesenchymal stem cells and progenitors need to adhere *in vitro*, and how it is influenced by FBS growth factors concentrations. Further studies will be necessary as progenitors adhesion represents one crucial step when it comes to mesenchymal progenitors isolation.

## Perspective

The fibroblast*-like* colony-forming assay is simple to perform: cells are plated; progenitors adhere to the substrate and proliferate in a short culture period; cells are fixed and stained; colonies with more than 50 cells are counted. The number of progenitors in a given cell population can be easily approximated. Colony-forming efficiency results, however, depend upon an optimized procedure and several steps must be optimized to provide linear and reproducible data. Herein, our purpose was to revisit this classic method and open a discussion based on three issues to assist assay performance and data interpretation, but mostly to help researchers establish in their labs a standardized procedure. It is critical to optimize the ability of the cells to proliferate in response to growth factors present in the FBS, or any other source used, and this is commonly neglected due to the “quantification-only” use of the method. As a renewable source for tissue regeneration, *in vitro* cell expansion is almost always required for MSC populations, meaning that these cells will not be used as primary cultures, but only after, at least, 3 passages. It would be expected that after several rounds of expansion, most primitive progenitors and stem cells would take over the culture at the expense of other progenitors. Nevertheless, this has not been well elucidated so far and it is not known how mesenchymal population responds to different growth factors concentration in different serum lots. Although several research groups have described different strategies MSCs, the cell populations obtained and expanded remain heterogeneous in terms of stem cells and committed progenitors regardless of the method used for isolation (Muraglia et al., 2000; Sacchetti et al., 2007). No current method ensures isolation of a pure population of MSCs, or even whether this is desirable but this fact, the lack of standards, makes difficult understanding of the biology and true potential of these cells.

**Figure 4:**
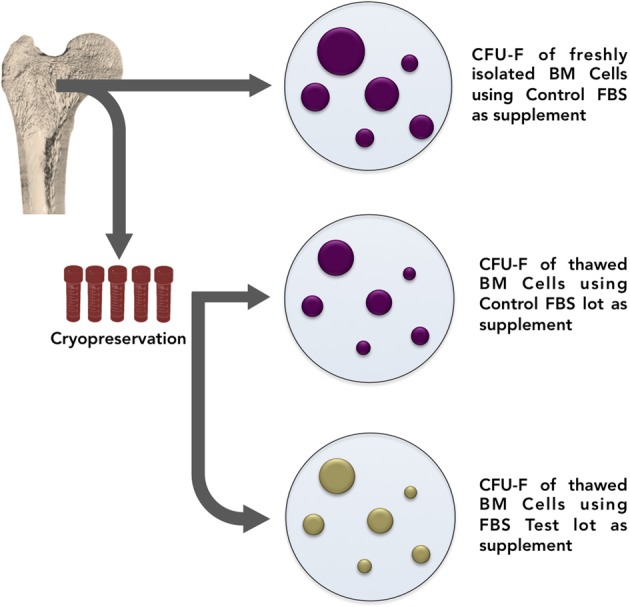
**In order to establish a “surveillance system” when working with mesenchymal progenitors isolation and expansion, it is suggested that each laboratory keep a standard collection of bone marrow samples to be used as references for testing serum lots.** Conceptually, each lab could select representative bone marrow samples, split each of them into equal aliquots (10, 20, 30, or as many as possible), freeze all at the same time, and cryopreserve. These aliquots will then be thawed and used to test serum “quality” every time changes of lots are necessary. From time to time, bone marrow collection must be renewed.

For these reasons it would be interesting that researchers establish a “surveillance system” when working with mesenchymal progenitors isolation and expansion. We therefore propose a list of three procedures recommended as controls. First, it would be highly desirable for each laboratory to keep a standard collection of bone marrow samples to be used as references for testing serum lots. Conceptually, each lab could select representative bone marrow samples, split each of them into equal aliquots (10, 20, 30, or as many as possible), freeze all at the same time, and cryopreserve (Figure 4). These aliquots will be used to test serum “quality” every time changes of lots are necessary.

**Figure 5:**
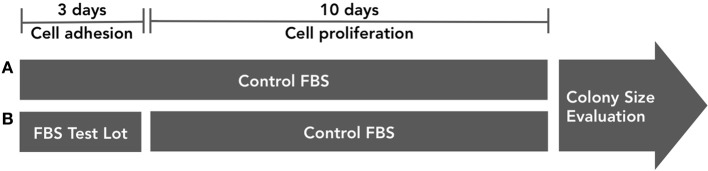
**FBS influence on progenitor cell adhesion *in vitro* must be tested.** In group **(A)**, bone marrow cells are incubated in culture medium supplemented with control serum. After 72 h, non-adherent cells are discarded and adherent cells are cultured for 10 additional days also in culture medium supplemented with control FBS. In group **(B)**, bone marrow cells are incubated in culture medium supplemented with testing serum. After 72 h, non-adherent cells are discarded and adherent cells are culture for 10 additional days in culture medium supplemented with control FBS. Colony numbers and size must be evaluated.

As a second control instrument, the influence of FBS in progenitors adhesion should be evaluated, regarding colony forming efficiency (Figure 5). In one group (we suggest triplicate), bone marrow cells are incubated in culture medium supplemented with control serum. After 72 h, non-adherent cells are discarded and adherent cells are cultured for 10 additional days also in culture medium supplemented with control FBS. In another group, bone marrow cells are incubated in culture medium supplemented with testing serum. After 72 h, non-adherent cells are discarded and adherent cells are culture for 10 additional days in culture medium supplemented with control FBS. The main objective of the assay is to analyze if different serum lots contribute to colony forming efficiency change at the expense of cell adhesion or cell proliferation.

**Figure 6:**
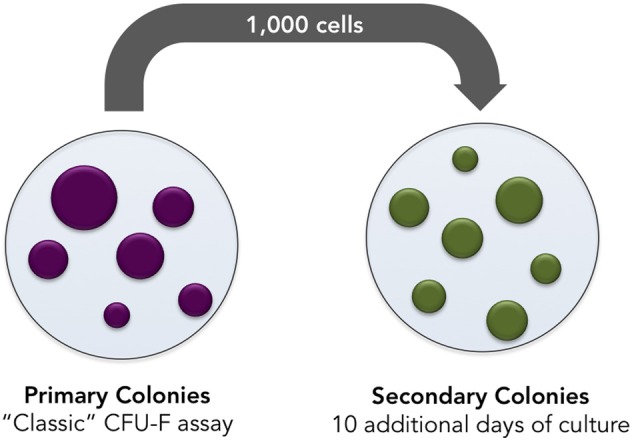
**Secondary colonies assay.** Bone marrow cells are incubated in culture medium supplemented with control or testing FBS. Seventy-two hours later, non-adherent cells are discarded and adherent cells are cultured in culture medium supplemented with control or testing serum for 10 additional days. Later, cells are tripsinized and single-cell suspension prepared. One thousand cells are subsequently replated and incubated for 10 additional days in culture medium supplemented with testing or control serum. Numbers and size of secondary colonies must be evaluated.

Mesenchymal progenitors' self-renewal capacity can be tested by evaluating the ability of expanded clones to originate secondary colonies upon replating. As a third parameter, we propose the secondary colony forming efficiency assay (Figure 6). One thousand cells from the primary colony-forming cultures may be replated and cultured for additional 10 days. Cells are, then, fixed and stained. It is important to highlight that, in all three assays colony numbers, and colony size must be rigorously evaluated and compared.

It is clear that cell behavior *in vivo* and *in vitro* changes from person to person, from animal to animal, even among syngeneic siblings, and over time in the same individual. We propose that these three strategies combining in-colony proliferation rate analyses with colonies formation and quantification, progenitors adhesion, and recloning ability, if used as a lab surveillance instrument, will assist in the development of a greater understanding of the biology of MSCs and other adherent populations *in vitro*. We would like to propose the creation of control guidelines which will facilitate the identification and isolation of long-term stem cells and short-term progenitors as a crucial step to better explore their potential and define their applicability in the cell therapy and bioengineering fields.

### Conflict of interest statement

The authors declare that the research was conducted in the absence of any commercial or financial relationships that could be construed as a potential conflict of interest.
